# 

**Published:** 1865-06

**Authors:** 


### CHICAGO MEDICAL JOURNAL.

**Terms, $2.00 per Annum.**

### CONTENTS OF THE JUNE NUMBER.

PAGE.

ORIGINAL COMMUNICATIONS.

On Diarrhœa, as seen in the Camps. By Erwin L. Jones, Ass't. Surg. 53d Wis. Inf. ........ 241

Clinical Lectures on Diseases of the Eye. By E. L. Holmes, M. D., of Chicago, Lecturer on Diseases of the Eye and Ear, in Rush Med. Col., etc.—The Sclerotica, or Sclera ........ 246

SELECTED.

Remarks on Dialysis. By Wm. Proctor, Jr. ........ 251

Sickness in Europe ........ 254

Uses of Glycerine ........ 259

Transformation of Muscular Fibre into Fat ........ 261

Influence of a Long Course of Nitric Acid in Reducing the Enlargement of the Liver and Spleen, that sometimes results from the Syphilitic Cachexy ........ 262

Lecture on the Results of the Surgical Treatment of Cancer. By T. Spencer Wells, F. R. C. S., Surgeon in ordinary to Her Majesty's Household ........ 263

Treatment of Acute Rheumatism ........ 270

Editorial and Miscellaneous.—Proceedings of the Illinois State Medical Society for 1865 ........ 275

Book Notices and Reviews ........ 288

